# Supplementary material for: Omega-3 Supplementation and Nutritional Status in Patients with Pancreatic Neoplasms: A Systematic Review
Source: Nutrients. 2024 Nov 26;16(23):4036. doi: 10.3390/nu16234036 (PMC11643750; doi:10.3390/nu16234036)
Supplement: Supplementary file 1 [file nutrients-16-04036-s001.zip › nutrients-3318728-supplementary.pdf]

Supplementary Material S1. Search strategy.

**MEDLINE/ PubMed**	#**1** (Adult[mesh terms] OR Adults[tiab] OR Young Adult[mesh terms] OR Adult, Young[tiab] OR Adults, Young[tiab] OR Young Adults[tiab] OR Middle Aged[mesh terms] OR Middle Age[tiab] OR "Aged"[mesh terms] OR Elderly[tiab]OR Aged, 80 and over[mesh terms] OR Oldest Old[tiab])<br><br>#**2** (Pancreatic Neoplasms[mesh terms] OR Neoplasm, Pancreatic[tiab] OR Pancreatic Neoplasm[tiab] OR Pancreas Neoplasms[tiab] OR Neoplasm, Pancreas[tiab] OR Neoplasms, Pancreas [tiab] OR Pancreas Neoplasm[tiab] OR Neoplasms, Pancreatic[tiab] OR Cancer of Pancreas[tiab] OR Pancreas Cancers[tiab] OR Pancreas Cancer[tiab] OR Cancer, Pancreas[tiab] OR Cancers, Pancreas[tiab] OR Pancreatic Cancer[tiab] OR Cancer, Pancreatic[tiab] OR Cancers, Pancreatic[tiab] OR Pancreatic Cancers[tiab] OR Cancer of the Pancreas[tiab] OR Pancreatic cancer, adult[tiab])<br><br>#**3** #1 AND #2<br><br>#**4** (Fatty Acids, Omega-3[mesh terms] OR Omega-3 Fatty Acid[tiab] OR Acid, Omega-3 Fatty[tiab] OR Fatty Acid, Omega-3[tiab] OR Omega 3 Fatty Acid[tiab] OR Omega-3 Fatty Acids[tiab] OR n-3 Oil[tiab] OR Oil, n-3[tiab] OR n 3 Oil[tiab] OR Oil, n3[tiab] OR n-3 Fatty Acids[tiab] OR n 3 Fatty Acids[tiab]OR Omega 3 Fatty Acids[tiab] OR n-3 PUFA[tiab] OR PUFA, n-3[tiab] OR n 3 PUFA[tiab] OR n3 Fatty Acid[tiab] OR Fatty Acid, n3[tiab] OR n3 PUFA[tiab] OR PUFA, n3[tiab] OR n3 Polyunsaturated Fatty Acid[tiab] OR n-3 Oils[tiab] OR n 3 Oils[tiab] OR N-3 Fatty Acid[tiab] OR Acid, N-3 Fatty[tiab] OR Fatty Acid, N-3[tiab] OR N 3 Fatty Acid[tiab] OR n-3 Polyunsaturated Fatty Acid[tiab] OR n 3 Polyunsaturated Fatty Acid[tiab] OR Eicosapentaenoic Acid[mesh terms] OR 5,8,11,14,17-Eicosapentaenoic Acid[tiab] OR Eicosapentanoic Acid[tiab] OR Acid, Eicosapentanoic[tiab] OR omega-3-Eicosapentaenoic Acid[tiab] OR omega 3 Eicosapentaenoic Acid[tiab] OR Timnodonic Acid[tiab] OR Icosapent[tiab] OR 5,8,11,14,17-Icosapentaenoic Acid[tiab] OR Docosahexaenoic Acids[mesh terms] OR Acids, Docosahexaenoic[tiab]OR Docosahexenoic Acids[tiab] OR Acids, Docosahexenoic[tiab] OR Docosahexaenoic Acid[tiab] OR Acid, Docosahexaenoic[tiab]OR Docosahexaenoic Acid[tiab] OR Acid, Docosahexaenoic[tiab] OR Docosahexaenoate[tiab] OR alpha-Linolenic Acid[mesh terms] OR Linolenic Acid[tiab])<br><br>#**5** #3 AND 4<br><br>#**6** (randomized controlled trial[pt] OR controlled clinical trial[pt] OR randomized[tiab] OR placebo[tiab] OR clinical trials as topic[mesh:noexp] OR randomly[tiab] OR trial[ti] NOT (animals[mh] NOT humans[mh]))<br><br>#**7** #5 AND #6
	#**1** ("Adult" OR "Adults" OR "Young Adult" OR "Adult, Young" OR "Adults, Young" OR "Young Adults" OR "Middle Aged" OR "Middle Age" OR "Aged" OR "Elderly" OR "Aged, 80 and over" OR "Oldest Old")

**CENTRAL Cohcrane**	#**2** (“Pancreatic Neoplasms” OR “Neoplasm, Pancreatic” OR “Pancreatic Neoplasm” OR “Pancreas Neoplasms” OR “Neoplasm, Pancreas” OR “Neoplasms, Pancreas” OR “Pancreas Neoplasm” OR “Neoplasms, Pancreatic” OR “Cancer of Pancreas” OR “Pancreas Cancers” OR “Pancreas Cancer” OR “Cancer, Pancreas” OR “Cancers, Pancreas” OR “Pancreatic Cancer” OR “Cancer, Pancreatic” OR “Cancers, Pancreatic” OR “Pancreatic Cancers” OR “Cancer of the Pancreas” OR “Pancreatic cancer, adult”)<br><br>#**3** #1 AND #2<br><br>#**4** **(**Fatty Acids, Omega-3 OR Omega-3 Fatty Acid OR Acid, Omega-3 Fatty OR Fatty Acid, Omega-3 OR Omega 3 Fatty Acid OR Omega-3 Fatty Acids OR n-3 Oil OR Oil, n-3 OR n 3 Oil OR Oil, n3 OR n-3 Fatty Acids OR n 3 Fatty Acids OR Omega 3 Fatty Acids OR n-3 PUFA OR PUFA, n-3 OR n 3 PUFA OR n3 Fatty Acid OR Fatty Acid, n3 OR n3 PUFA OR PUFA, n3 OR n3 Polyunsaturated Fatty Acid OR n-3 Oils OR n 3 Oils OR N-3 Fatty Acid OR Acid, N-3 Fatty OR Fatty Acid, N-3 OR N 3 Fatty Acid OR n-3 Polyunsaturated Fatty Acid OR n 3 Polyunsaturated Fatty Acid)<br><br>#**5** #3 AND #4
**EMBASE**	#**1**(('adult'/exp OR 'adult':ti,ab OR 'adults':ti,ab OR 'middle aged'/exp OR 'middle age':ti,ab OR 'middle aged':ti,ab OR 'young adult'/exp OR 'adult, young':ti,ab OR 'young adult':ti,ab OR 'young adults':ti,ab OR 'aged'/exp OR 'aged':ti,ab OR 'aged patient':ti,ab OR 'aged people':ti,ab OR 'aged person':ti,ab OR 'aged subject':ti,ab OR 'elderly':ti,ab OR 'elderly patient':ti,ab OR 'elderly people':ti,ab OR 'elderly person':ti,ab OR 'elderly subject':ti,ab OR 'senior citizen':ti,ab OR 'senium':ti,ab)<br><br>#**2** ('pancreas tumor'/exp OR 'neoplasia of the pancreas':ti,ab OR 'neoplasm of the pancreas':ti,ab OR 'neoplastic pancreas':ti,ab OR 'neoplastic pancreatic':ti,ab OR 'pancreas neoplasia':ti,ab OR 'pancreas neoplasm':ti,ab OR 'pancreas tumor':ti,ab OR 'pancreas tumorigenesis':ti,ab OR 'pancreas tumour':ti,ab OR 'pancreatic neoplasm':ti,ab OR 'pancreatic neoplasms':ti,ab OR 'pancreatic tumor':ti,ab OR 'pancreatic tumorigenesis':ti,ab OR 'pancreatic tumour':ti,ab OR 'tumor of the pancreas':ti,ab OR 'tumour of the pancreas':ti,ab) OR 'pancreatic neoplasms':ti,ab)<br><br>#**3** ('omega 3 fatty acid'/exp OR 'bilantin omega':ti,ab OR 'eicosa e':ti,ab OR 'eicosapen':ti,ab OR 'epaisdin':ti,ab OR 'epanova':ti,ab OR 'fatty acids, omega 3':ti,ab OR 'n 3 fatty acid':ti,ab OR 'n 3 polyunsaturated fatty acid':ti,ab OR 'omega 3':ti,ab OR 'omega 3 carboxylic acid':ti,ab OR 'omega 3 carboxylic acids':ti,ab OR 'omega 3 fatty acid':ti,ab OR 'omega 3 feingold':ti,ab OR 'omega 3 plus':ti,ab OR 'omega 3 polyunsaturated fatty acid':ti,ab OR 'omega forte':ti,ab OR 'omega-3-carboxylic acids':ti,ab OR 'omega3 polyunsaturated fatty acid':ti,ab OR 'sanhelios omega 3':ti,ab OR 'fatty acids, omega-3':ti,ab)<br><br>#**4** #1 AND #2 AND #3
	#**1** TS=((“Adult” OR “Adults” OR “Young Adult” OR “Adult, Young” OR “Adults, Young” OR “Young Adults” OR “Middle Aged” OR “Middle Age” OR “Aged” OR “Elderly” OR “Aged, 80 and over” OR “Oldest Old”))<br><br>#**2** TS=((“Pancreatic Neoplasms” OR “Neoplasm, Pancreatic” OR “Pancreatic Neoplasm” OR “Pancreas Neoplasms” OR “Neoplasm, Pancreas” OR “Neoplasms,

**Web of Science**	Pancreas" OR "Pancreas Neoplasm" OR "Neoplasms, Pancreatic" OR "Cancer of Pancreas" OR "Pancreas Cancers" OR "Pancreas Cancer" OR "Cancer, Pancreas" OR "Cancers, Pancreas" OR "Pancreatic Cancer" OR "Cancer, Pancreatic" OR "Cancers, Pancreatic" OR "Pancreatic Cancers" OR "Cancer of the Pancreas" OR "Pancreatic cancer, adult"))<br><br>#**3** TS=(("Fatty Acids, Omega-3" OR "Omega-3 Fatty Acid" OR "Acid, Omega-3 Fatty" OR "Fatty Acid, Omega-3" OR "Omega 3 Fatty Acid" OR "Omega-3 Fatty Acids" OR "n-3 Oil" OR "Oil, n-3" OR "n 3 Oil" OR "Oil, n3" OR "n-3 Fatty Acids" OR "n 3 Fatty Acids" OR "Omega 3 Fatty Acids" OR "n-3 PUFA" OR "PUFA, n-3" OR "n 3 PUFA" OR "n3 Fatty Acid" OR "Fatty Acid, n3" OR "n3 PUFA" OR "PUFA, n3" OR "n3 Polyunsaturated Fatty Acid" OR "n-3 Oils" OR "n 3 Oils" OR "N-3 Fatty Acid" OR "Acid, N-3 Fatty" OR "Fatty Acid, N-3" OR "N 3 Fatty Acid" OR "n-3 Polyunsaturated Fatty Acid" OR "n 3 Polyunsaturated Fatty Acid"))_<br><br>#**4** #1 AND #2 AND #3
**Scopus**	#**1** TITLE-ABS-KEY(("Adult" OR "Adults" OR "Young Adult" OR "Adult, Young" OR "Adults, Young" OR "Young Adults" OR "Middle Aged" OR "Middle Age" OR "Aged" OR "Elderly" OR "Aged, 80 and over" OR "Oldest Old"))<br><br>#**2** TITLE-ABS-KEY(("Pancreatic Neoplasms" OR "Neoplasm, Pancreatic" OR "Pancreatic Neoplasm" OR "Pancreas Neoplasms" OR "Neoplasm, Pancreas" OR "Neoplasms, Pancreas" OR "Pancreas Neoplasm" OR "Neoplasms, Pancreatic" OR "Cancer of Pancreas" OR "Pancreas Cancers" OR "Pancreas Cancer" OR "Cancer, Pancreas" OR "Cancers, Pancreas" OR "Pancreatic Cancer" OR "Cancer, Pancreatic" OR "Cancers, Pancreatic" OR "Pancreatic Cancers" OR "Cancer of the Pancreas" OR "Pancreatic cancer, adult"))<br><br>#**3** TITLE-ABS-KEY(("Fatty Acids, Omega-3" OR "Omega-3 Fatty Acid" OR "Acid, Omega-3 Fatty" OR "Fatty Acid, Omega-3" OR "Omega 3 Fatty Acid" OR "Omega-3 Fatty Acids" OR "n-3 Oil" OR "Oil, n-3" OR "n 3 Oil" OR "Oil, n3" OR "n-3 Fatty Acids" OR "n 3 Fatty Acids" OR "Omega 3 Fatty Acids" OR "n-3 PUFA" OR "PUFA, n-3" OR "n 3 PUFA" OR "n3 Fatty Acid" OR "Fatty Acid, n3" OR "n3 PUFA" OR "PUFA, n3" OR "n3 Polyunsaturated Fatty Acid" OR "n-3 Oils" OR "n 3 Oils" OR "N-3 Fatty Acid" OR "Acid, N-3 Fatty" OR "Fatty Acid, N-3" OR "N 3 Fatty Acid" OR "n-3 Polyunsaturated Fatty Acid" OR "n 3 Polyunsaturated Fatty Acid"))<br><br>#**4** #1 AND #2 AND #3

Supplementary Material S2. Full articles excluded (n = 19) of systematic review with reasons.

Reference	Reasons*
Arshad, A. et al., 2014	3
Barber, M. D. et al., 2004	3
Davidson, W. et al., 2004	4
Di Carlo, V. et al., 1999	2
Gade, J. et al., 2016	3
Gumpper-Fedus, K. et al., 2022	4
Hager, E. D. et al., 2009	3
Isherwood, J. et al., 2020	3
Klek, S. et al., 2008	1
Macášek, J. et al., 2012	4
Martin Ii, R. C. G. et al., 2017	2
Matejcic, M. et al., 2018	4
Morales, E. et al., 2007	4
Moses, A. W. G. et al., 2004	3
Nakamura, K. et al., 2005	1
Ramalho, R. Et al., 2017	4
Shishavan, N. G. et al., 2021	4
Suzuki, D. et al., 2010	1
Turunen, A. W. et al., 2014	4

*Reasons for excluding complete articles: 1) Wrong population; 2) Intervention with immunonutrition; 3) Primary outcome; 4) Study design.

References

1. Arshad, A. et al. Restoration of mannose-binding lectin complement activity is associated with improved outcome in patients with advanced pancreatic cancer treated with gemcitabine and intravenous ω-3 fish oil. **J. Parent.Enteral Nutrition**, v. 38, n. 2, p. 214-219, 2014.
2. Barber, M. D. et al. Modulation of the liver export protein synthetic response to feeding by an n− 3 fatty-acid-enriched nutritional supplement is associated with anabolism in cachectic cancer patients. **Clinical science**, v. 106, n. 4, p. 359-364, 2004.
3. Davidson, W. et al. Weight stabilisation is associated with improved survival duration and quality of life in unresectable pancreatic cancer. **Clinical nutrition**, v. 23, n. 2, p. 239-247, 2004.
4. Di Carlo, V. et al. Complications of pancreatic surgery and the role of perioperative nutrition. **Digestive surgery**, v. 16, n. 4, p. 320-326, 1999.
5. Gade, J. et al. The effect of preoperative oral immunonutrition on complications and length of hospital stay after elective surgery for pancreatic cancer–a randomized controlled trial. **Nutrition and Cancer**, v. 68, n. 2, p. 225-233, 2016.
6. Gumpper-Fedus, K. et al. Altered Plasma Fatty Acid Abundance Is Associated with Cachexia in Treatment-Naïve Pancreatic Cancer. **Cells**, v. 11, n. 5, p. 910, 2022.
7. Hager, E. D. et al. Multimodale komplementäre therapie des fortgeschrittenen pankreaskarzinoms. **Deutsche Zeitschrift für Onkologie**, v. 41, n. 01, p. 16-26, 2009.
8. Isherwood, J. et al. Myeloid derived suppressor cells are reduced and T regulatory cells stabilised in patients with advanced pancreatic cancer treated with gemcitabine and intravenous omega 3. **Annals of Translational Medicine**, v. 8, n. 5, 2020.
9. Klek, S. et al. Standard and immunomodulating enteral nutrition in patients after extended gastrointestinal surgery–a prospective, randomized, controlled clinical trial. **Clinical nutrition**, v. 27, n. 4, p. 504-512, 2008.
10. Macášek, J. et al. Plasma fatty acid composition in patients with pancreatic cancer: correlations to clinical parameters. **Nutrition and cancer**, v. 64, n. 7, p. 946-955, 2012.
11. Martin Ii, R. C. G. et al. Efficacy of preoperative immunonutrition in locally advanced pancreatic cancer undergoing irreversible electroporation (IRE). **European Journal of Surgical Oncology (EJSO)**, v. 43, n. 4, p. 772-779, 2017.
12. Matejcic, M. et al. Circulating plasma phospholipid fatty acids and risk of pancreatic cancer in a large European cohort. **International journal of cancer**, v. 143, n. 10, p. 2437-2448, 2018.

13. Morales, E. et al. Food and nutrient intakes and K-ras mutations in exocrine pancreatic cancer. **Journal of Epidemiology & Community Health**, v. 61, n. 7, p. 641-649, 2007.
14. Moses, A. W. G. et al. Reduced total energy expenditure and physical activity in cachectic patients with pancreatic cancer can be modulated by an energy and protein dense oral supplement enriched with n-3 fatty acids. **British journal of cancer**, v. 90, n. 5, p. 996-1002, 2004.
15. Nakamura, K. et al. Influence of preoperative administration of ω-3 fatty acid-enriched supplement on inflammatory and immune responses in patients undergoing major surgery for cancer. **Nutrition**, v. 21, n. 6, p. 639-649, 2005.
16. Ramalho, R. et al. Omega-3 therapeutic supplementation in a patient with metastatic adenocarcinoma of the pancreas with muscle mass depletion. **European Journal of Clinical Nutrition**, v. 71, n. 6, p. 795-797, 2017.
17. Shishavan, N. G. et al. Circulating plasma fatty acids and risk of pancreatic cancer: Results from the Golestan Cohort Study. **Clinical Nutrition**, v. 40, n. 4, p. 1897-1904, 2021.
18. Suzuki, D. et al. Effects of perioperative immunonutrition on cell-mediated immunity, T helper type 1 (Th1)/Th2 differentiation, and Th17 response after pancreaticoduodenectomy. **Surgery**, v. 148, n. 3, p. 573-581, 2010.
19. Turunen, A. W. et al. Cancer incidence in a cohort with high fish consumption. **Cancer Causes & Control**, v. 25, n. 12, p. 1595-1602, 2014.
